# Availability of Nitrite and Nitrate as Electron Acceptors Modulates Anaerobic Toluene-Degrading Communities in Aquifer Sediments

**DOI:** 10.3389/fmicb.2020.01867

**Published:** 2020-08-14

**Authors:** Baoli Zhu, Sebastian Friedrich, Zhe Wang, András Táncsics, Tillmann Lueders

**Affiliations:** ^1^Chair of Ecological Microbiology, University of Bayreuth, Bayreuth, Germany; ^2^Chair of Hydrogeology, Technical University of Munich, Munich, Germany; ^3^Regional University Center of Excellence in Environmental Industry, Szent Istvan University, Gödöllö, Hungary

**Keywords:** toluene degradation, bioremediation, stable isotope probing, oxygenic denitrification, *Azoarcus*

## Abstract

Microorganisms are essential in the degradation of environmental pollutants. Aromatic hydrocarbons, e.g., benzene, toluene, ethylbenzene, and xylene (BTEX), are common aquifer contaminants, whose degradation *in situ* is often limited by the availability of electron acceptors. It is clear that different electron acceptors such as nitrate, iron, or sulfate support the activity of distinct degraders. However, this has not been demonstrated for the availability of nitrate vs. nitrite, both of which can be respired in reductive nitrogen cycling. Here *via* DNA-stable isotope probing, we report that nitrate and nitrite provided as electron acceptors in different concentrations and ratios not only modulated the microbial communities responsible for toluene degradation but also influenced how nitrate reduction proceeded. Zoogloeaceae members, mainly *Azoarcus* spp., were the key toluene degraders with nitrate-only, or both nitrate and nitrite as electron acceptors. In addition, a shift within *Azoarcus* degrader populations was observed on the amplicon sequence variant (ASV) level depending on electron acceptor ratios. In contrast, members of the Sphingomonadales were likely the most active toluene degraders when only nitrite was provided. Nitrate reduction did not proceed beyond nitrite in the nitrate-only treatment, while it continued when nitrite was initially also present in the microcosms. Likely, this was attributed to the fact that different microbial communities were stimulated and active in different microcosms. Together, these findings demonstrate that the availability of nitrate and nitrite can define degrader community selection and N-reduction outcomes. It also implies that nitrate usage efficiency in bioremediation could possibly be enhanced by an initial co-supply of nitrite, *via* modulating the active degrader communities.

## Introduction

Aromatic hydrocarbons like benzene, toluene, ethylbenzene, and xylene (BTEX) are among the most common pollutants threatening the quality of groundwater and aquifer ecosystem status (Lueders, 2017). Since BTEX compounds are carcinogenic and neurotoxic to humans, and it is important to understand their behavior and fate in groundwater systems and to develop feasible and effective remediation strategies. Microorganisms can degrade hydrocarbons, including BTEX compounds, aerobically or phototrophically. In environments where the oxygen availability is limited, hydrocarbons can also be degraded anaerobically, carried out by microbes using nitrate, nitrite, sulfate, or iron (III) as electron acceptors or by methanogenic consortia (Rabus et al., 2016). Under anoxic conditions, nitrate and sulfate are the most important soluble electron acceptors used by anaerobic hydrocarbon-degraders in freshwater and marine environments, and a considerable number of enrichments and strains have been cultivated and characterized from different habitats (Widdel et al., 2010). Nearly all denitrifying hydrocarbon-degrading cultures have been obtained using nitrate as electron acceptor. Many of these cultures reduce nitrate *via* nitrite to nitrogen gas (N_2_); some others, such as *Geobacter metallireducens* form ammonia from nitrate (Lovley et al., 1993). There are also cultures reported to only produce and accumulate nitrite, e.g., the naphthalene-degrading *Vibrio pelagius* and the biphenyl-degrading *Citrobacter freundii* (Rockne et al., 2000; Grishchenkov et al., 2002).

Nitrate amendment is a frequently used strategy for enhancing bioremediation *in-situ*, due to its high solubility and ability to support a wide spectrum of hydrocarbon-degrading microbes. For instance, controlled-release of nitrate enhanced phenanthrene-degradation in undisturbed marine sediments, likely through stimulating the indigenous degraders (Tang et al., 2005). Injection of nitrate into a crude oil-contaminated aquifer effectively decreased dissolved hydrocarbon concentrations, including benzene, within 3 months (Ponsin et al., 2014). In anoxic laboratory microcosms of gasoline-impacted soil, nitrate amendment also stimulated toluene, ethylbenzene, and total petroleum hydrocarbon degradation (Yang et al., 2016). However, microbial community responses to such electron acceptor amendments are typically complex and often not well-documented. A study employing functional gene-arrays has reported that, besides N-cycling genes, functional genes involved in carbon-, sulfur-, and phosphorus-cycling were also enriched after nitrate injection to a hydrocarbon contaminated estuarine sediment (Xu et al., 2014), complicating the prediction of specific impacts of electron acceptor amendment.

Nitrate reduction routes can be influenced by many factors, including concentrations of available nitrate and hydrocarbons, indigenous microbial groups, as well as many other environmental parameters (Kraft et al., 2014). Nitrite, as an obligate intermediate of all nitrate reduction pathways, has been reported to transiently accumulate both in laboratory cultures and at bioremediation sites (Kelso et al., 1999; Chayabutra and Ju, 2000; Ponsin et al., 2014). However, nitrite alone or in combination with nitrate is rarely applied in bioremediation, and the impact of nitrite as an electron acceptor on hydrocarbon degradation processes and the selection of active degraders has not been well-investigated. Nevertheless, for denitrifying anaerobic methane oxidizers, it is clear that the concentrations of nitrate and nitrite provided strongly influence the selection and activity of different key players. When similar concentrations of nitrate and nitrite were present as electron acceptors, both methanotrophic archaea, *Methanoperedens* spp., and NC10 bacteria (Rokubacteria) were enriched and active in methane oxidation (Raghoebarsing et al., 2006). Whereas nitrate alone supports the enrichment of *Methanoperedens* spp., nitrite favors the activity and growth of NC10 bacteria (Ettwig et al., 2008, 2016; Haroon et al., 2013). In fact, the NC10 bacterium “*Candidatus* Methylomirabilis oxyfera” is proposed to form its own intracellular oxygen during anaerobic growth with nitrite *via* NO-dismutation, a process termed oxygenic denitrification (Ettwig et al., 2010). Diverse oxygenic denitrifiers have also been reported from the BTEX-contaminated Siklós aquifer, however, whether they are involved in BTEX-biodegradation remains unclear (Zhu et al., 2017, 2019).

Nitrate is generally preferred in bioremediation, one reason is that by complete denitrification, the same molar amendment of nitrate has 67% more electron accepting capacity than nitrite, due to the different oxidation states of N (Kuypers et al., 2018). In addition, nitrite is very reactive and difficult to detect in the environment, and it may be also toxic to hydrocarbon-degrading microorganisms (Ulrich and Edwards, 2003). Nonetheless, in the environment, nitrate-driven hydrocarbon degradation hardly proceeds *via* the theoretical stoichiometry and is often incomplete. While oversupply of nitrate not only increases cost, but also can lead to other unwanted consequences, e.g., nitrite accumulation and N_2_O emissions. Thus, it is important to determine the optimal amount of nitrate needed for supporting *in situ* biodegradation and, at the same time, avoiding adverse effects of nitrite and nitrogen oxide accumulation. In this study, the influence of nitrate vs. nitrite availability as electron acceptors on the selection of active toluene degrading populations and the progression of nitrate reduction was investigated *via* a DNA-SIP experiment with ^13^C_7_-labeled toluene. Whether oxygenic denitrifiers were possibly involved in the process was also queried. The findings can help to better understand microbial responses to electron acceptor amendments and to optimize nitrate-dependent bioremediation strategies.

## Materials and Methods

### Sampling Site

Sediment samples were taken in April 2018 from the sump of a monitoring well (ST-2) of a BTEX-contaminated aquifer in Siklós, Hungary. Previous studies have shown that diverse hydrocarbon-degrading microorganisms are present and active in the aquifer (Tancsics et al., 2012, 2013). The sediment was retrieved as previously described (Tancsics et al., 2018).

### Microcosm Incubations With ^13^C_7_-Toluene

For each microcosm incubation, 5 ml homogenously mixed sediment slurry was transferred into sterile 100 ml serum bottles containing 50 ml of autoclaved artificial groundwater medium (Winderl et al., 2010). Incubations containing about 0.8 mM ^13^C_7_-toluene and different electron acceptor combinations were set up in triplicates. To ensure complete toluene oxidation, the total electron-accepting potential of nitrate and nitrite provided was about 50 mmol l^−1^. According to nitrate/nitrite concentrations provided, the microcosms were designated as the follows: 8/0 microcosm with sole nitrate (8 mM), 5/7 microcosm with nitrate (5 mM) and nitrite (7 mM). 2/12 microcosm with both nitrate (2 mM) and nitrite (12 mM), and 0/18 microcosm with sole nitrite (18 mM). Control microcosms provided with 1 mM unlabeled ^12^C-toluene and without electron acceptor amendment were used to exclude aerobic toluene degradation. All serum bottles were sealed with air tight stoppers and sparged aseptically with N_2_/CO_2_ (80:20, v/v) for 5 min. Toluene was then added by injection with a glass syringe after headspace sparging. The initial pH of all incubations was ~7.3. The bottles were incubated at room temperature on a shaker at 100 rpm for 11 days. To avoid unspecific labeling *via* cross feeding, one replicate of each treatment was sacrificed for sediment sampling on the 7th day, when most toluene was consumed in nearly all incubations. The remaining duplicate serum bottles were further incubated for one more week.

### Toluene, Nitrate, and Nitrite Quantification

Toluene concentrations were measured *via* headspace analysis on a Trace DSQ GC/MS (Thermo Electron, Germany) equipped with a Combi PAL autosampler (CTC Analytics, Switzerland) as previously described (Avramov et al., 2013). A DB5-MS capillary column (Agilent Technologies, Germany) and helium as carrier gas at a flow rate of 1 ml min^−1^ were used. Briefly, 1 ml of slurry was taken with glass syringe from each serum bottle and transferred to a 2 ml vial. Ethylbenzene was added with a glass syringe to a final concentration of 2.3 mg l^−1^ as internal standard, and then the vial was immediately capped. Standard vials containing different concentrations of toluene (0, 0.25, 0.5, 0.75, 1.0, and 1.5 mM) but with the same amount of internal standard were prepared in the same way. Both the sample and standard vials were shaken for 17 min at 70°C to reach toluene liquid-headspace equilibrium. Then, 100 μl of headspace gas was injected into the GC-MS. Toluene concentrations were calculated according to the calibration standard.

Liquid samples filtered through a 0.45 μm filter (Millex-GP; Merck Milipore, Germany) were used for nitrate and nitrite quantification with ion chromatography using a coupled Dionex ICS 1100 system, equipped with an AS4A 4 × 250 mm and a CS12 A 4 × 250 mm column for anions and cations, respectively.

### DNA Extraction and Ultracentrifugation

In order to identify which microbes were involved in toluene degradation by DNA-SIP, one representative incubation from all, except the 2/12 microcosms, was sacrificed for DNA extraction at day 7, when most of added toluene was degraded. Since the 5/7 and the 2/12 microcosms showed similar pattern of activity, only the 5/7 microcosm was used for representing the condition of both nitrate and nitrite present as electron acceptor. Sediment DNA was isolated as previously described (Pilloni et al., 2012) with minor modifications. The final DNA precipitation was done at 4°C instead of 20°C. About 0.4 g sediment (wet weight) was used for each DNA extraction, and two extractions were performed from each sample. DNA was quantified with the Quant-iT PicoGreen dsDNA Assay Kit (Thermo Fisher, Waltham, USA) on an MX3000p cycler (Agilent, Santa Clara, USA), and checked by standard agarose gel electrophoresis. Two DNA extracts from the same sample were pooled and stored at −20°C until gradient centrifugation.

Ultracentrifugation, fractionation and DNA recovery were performed as previously described (Lueders, 2015). Approximately 1 μg of DNA extract was loaded onto a gradient medium of CsCl (average density 1.72 g ml^−1^, Calbiochem, Darmstadt, Germany) in gradient buffer (0.1 M Tris-HCl at pH 8, 0.1 M KCl, and 1 mM EDTA). The sealed polyallomer tubes (QuickSeal, Beckman, California) were centrifuged at 44,500 rpm (184,000 g) for about 48 h as previously described (Lueders, 2015). A total of 12 fractions (each *ca*. 380–400 μl) from each gradient were collected from “heavy” to “light.” Refractory index of each fraction was measured with refractometer (DR201-95, Krüss, Hamburg, Germany), and buoyant density was calculated based on reference standards. DNA in gradient fractions were precipitated with two volume PEG, washed with 150 μl ice-cold 70% ethanol, and then resuspended in 25 μl elution buffer and stored at −20°C.

### qPCR and Amplicon Sequencing

The distribution of density-resolved DNA over gradient fractions was quantified *via* 16S rRNA gene qPCR using bacterial primers Ba519F and Ba907R (Winderl et al., 2008). To test for the potential involvement of oxygenic denitrifiers in toluene degradation, putative nitric oxide dismutase (*nod*) genes were also quantified over gradient fractions using primer pair nod1446F and nod1706Rv2 (Zhu et al., 2017). qPCR was performed as previously described (Zhu et al., 2020).

For each gradient, DNA from representative light and heavy fractions with highest 16S rRNA and *nod* gene counts were selected for amplicon sequencing. 16S rRNA gene V4 amplicons were generated with the universal 16S primer pair 515F and 806R (Walters et al., 2016) from selected light and heavy gradient fractions. Sequencing libraries were prepared with the Nextera XT v2 kit and were sequenced on an Illumina iSeq-100. The sequencing was done in the Genomics and Bioinformatics Key Lab of Bayreuth University.

### Sequencing Data Handling and Phylogenetic Analysis

Amplicon data were processed and analyzed with the DADA2 package in R, including quality filtering, denoizing, chimera removal, amplicon sequence variant (ASV) inference, and taxonomic assignment as previously described (Callahan et al., 2016, 2019). Abundant ASVs (≥2%) were aligned with reference 16S rRNA sequences with the ClustralW algorithm with default settings, and based on the alignment, a phylogenetic tree was constructed using the neighbor-joining method in MEGA-X. The robustness of the tree topology was tested by bootstrap analysis (1,000 replicates).

### Nucleotide Sequence Deposition

Sequencing data are available at NCBI with SRA accession: PRJNA631140.

## Results

### Toluene Degradation and Nitrate, Nitrite Reduction

**Figure 1:**
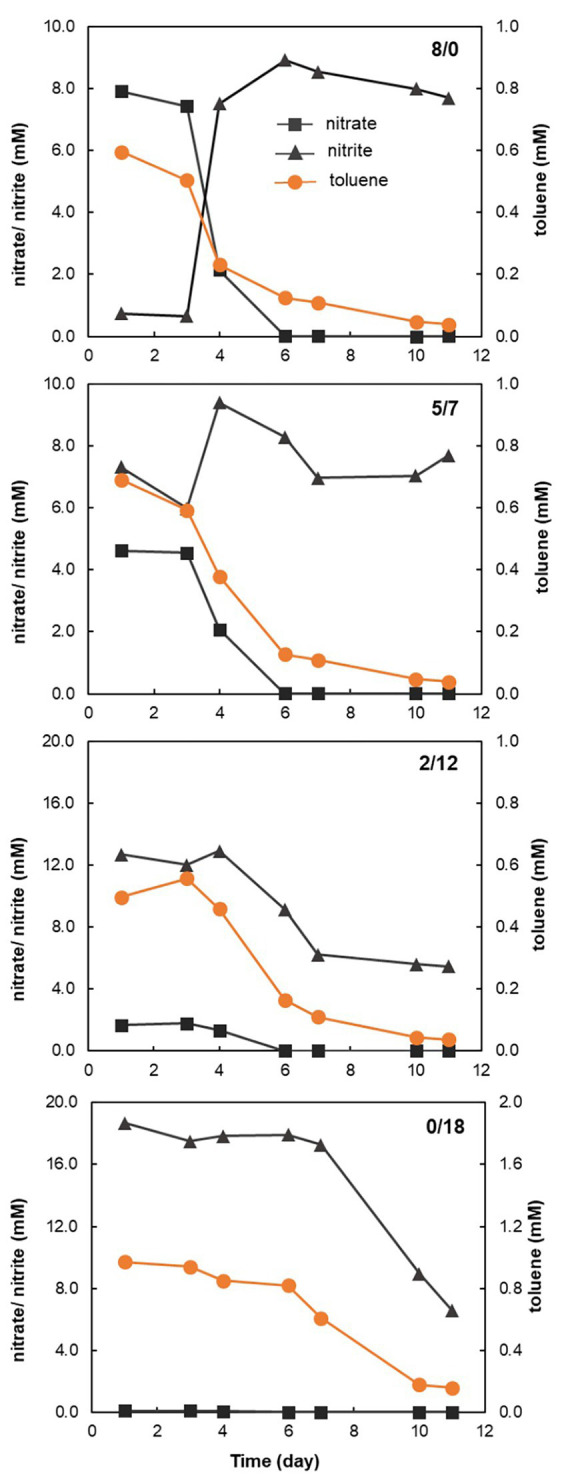
Activity of nitrate, nitrite reduction and toluene oxidation in all except the control treatment microcosms. The first 7 days data are the average of three replicate incubations, while the other data points are the average of the two rest incubations. The **8/0** panel represents the results of the microcosm contained 8/0 mM (nitrate/nitrite) as electron acceptors; the **5/7** panel represents the results of the microcosm contained 5/7 mM (nitrate/nitrite) as electron acceptors; the **2/12** panel represents the results of the microcosm contained 2/12 mM (nitrate/nitrite) as electron acceptors; and the **0/18** panel represents the results of the microcosm contained 0/18 mM (nitrate/nitrite) as electron acceptors.

For the 8/0, 5/7, and 2/12 treatments, toluene was largely degraded within one week (Figure 1). In the 0/18 microcosms, the onset of toluene degradation was delayed, only a small part of toluene was degraded in the first week. No loss of toluene was observed in the non-electron acceptor control microcosms (data not shown). In the 8/0 microcosms, all nitrate appeared to be reduced to an equal molar amount of nitrite, which accumulated over the incubation period (Figure 1). In the 5/7 microcosms, where both nitrate and nitrite were provided, it seemed that nitrite rather than nitrate was first reduced. Nitrate reduction only started on the 3rd day, with a concomitant accumulation of nitrite, which then was further reduced. However, at the end of the incubation, nitrite was close to its initial concentration of ~7 mM (Figure 1). Similarly, in the 2/12 microcosms, nitrite seemed to be reduced first, and nitrate reduction started on the 3rd day, leading to a minor increase of nitrite concentration. Then both nitrate and nitrite were consumed simultaneously. In contrast, nitrite reduction in the 0/18 microcosm was insignificant in the 1st week, both toluene and nitrite were only markedly reduced afterwards.

### Distribution of Total Bacteria and Oxygenic Denitrifiers in Density Gradients

**Figure 2:**
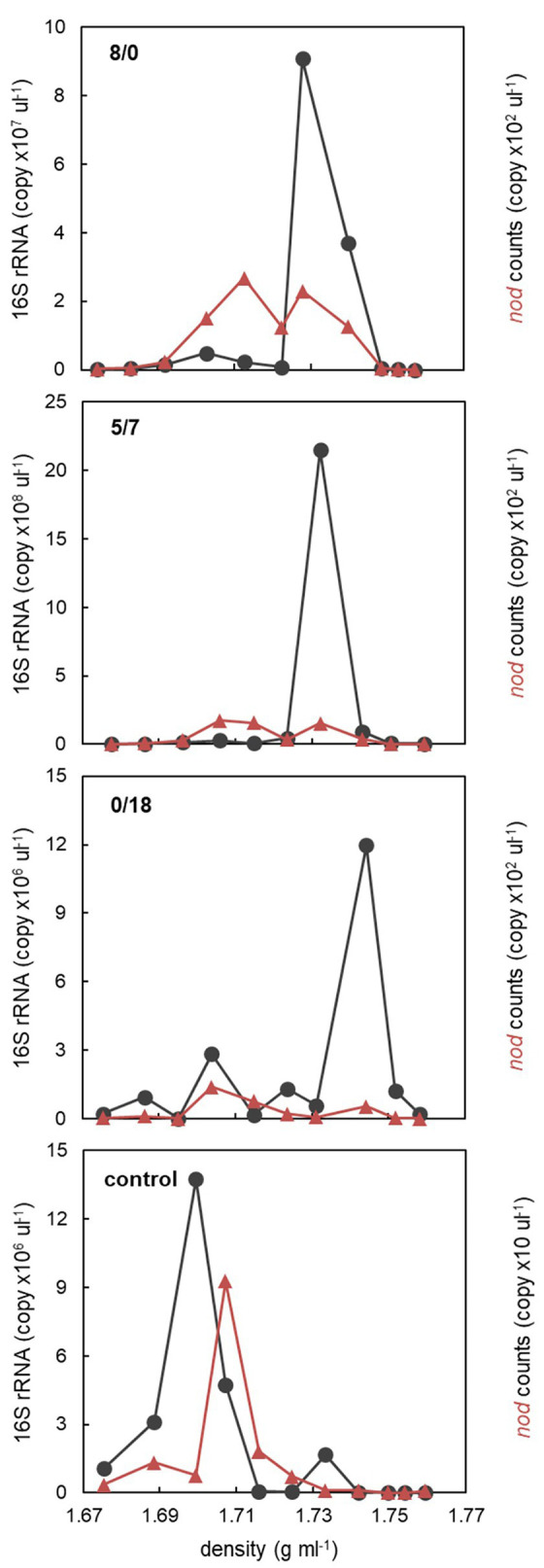
The abundance distribution of all bacteria (16S rRNA) and oxygenic denitrifiers (*nod*) along the density gradients of the 8/0, 5/7, 0/18, and the control microcosms. For each microcosm, DNA recovered from the fractions with highest 16S rRNA or *nod* counts at both light and heavy densities were used for amplicon sequencing analysis.

One microcosm from each of the 8/0, 5/7, 0/18, and control treatments was sacrificed on day 7 for DNA-SIP analysis. To check how DNA was allocated along SIP density gradients, 16S rRNA targeted qPCR was performed on DNA recovered from all fractions of the 8/0, 5/7, 0/18, and control microcosms. Except the control, two 16S rRNA abundance peaks were present in light and heavy fractions, respectively (Figure 2). Especially abundant peaks of heavy DNA indicative of efficient ^13^C-labeling were detected in heavy fractions between 1.73 and 1.74 g ml^−1^, in the 8/0 and 5/7 gradients. The 0/18 microcosm also showed a peak of bacterial qPCR counts at 1.73 g ml^−1^, but it was an order of magnitude lower compared to the previous treatments. In the control treatment, the peak abundance of DNA was detected in light fractions at 1.70 g ml^−1^, with a minor peak also tailing into heavier fractions. This indicated that ^13^C-labeling and density-based DNA separation was successful.

In order to check if oxygenic denitrifiers were also detectable in SIP gradients, putative *nod* genes were also quantified by qPCR in gradient fractions. In all ^13^C_7_-toluene microcosms, *nod* genes appeared to be distributed over two peaks across lighter and heavier fractions, but with a much lower absolute abundance (<2 × 10^2^ copy μl^−1^) compared to that of 16S rRNA. Only one *nod* peak was found in the light fractions of the control SIP gradient (Figure 2). This indicates that some *nod*-carrying microbes, if not all, also assimilated ^13^C from amended toluene.

### Identification of Labeled Bacteria by DNA-SIP

Representative light and heavy SIP fractions with high 16S rRNA gene counts were selected for amplicon sequencing. Eight amplicon libraries were sequenced, resulting in ≥50,000 reads for each library. After quality control and assembly, between ~34,000 and 54,000 nonchimeric reads remained. From these, 686 amplicon sequence variants (ASVs) were inferred in total. Only 34 of these ASVs were detected at a relative abundance ≥2% in at least one library.

**Figure 3:**
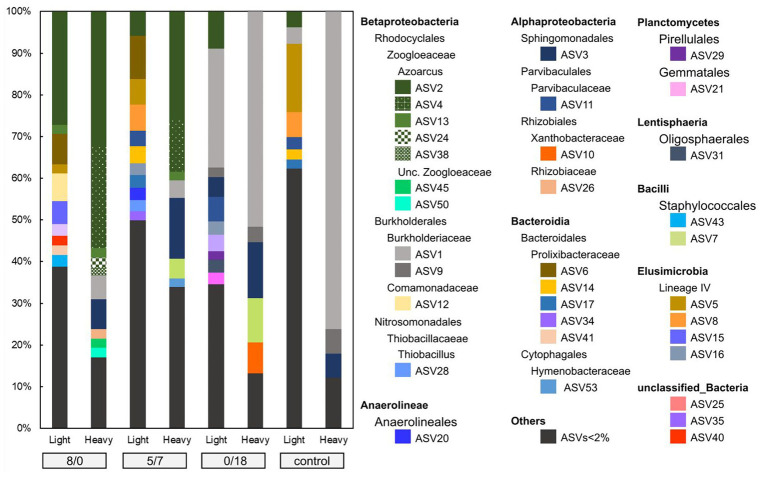
ASVs (amplicon sequencing variants) diversity in light and heavy fractions of the **8/0**, **5/7**, **0/18**, and the **control** microcosms. ASVs with relative abundance <2% were not individually displayed.

**Figure 4:**
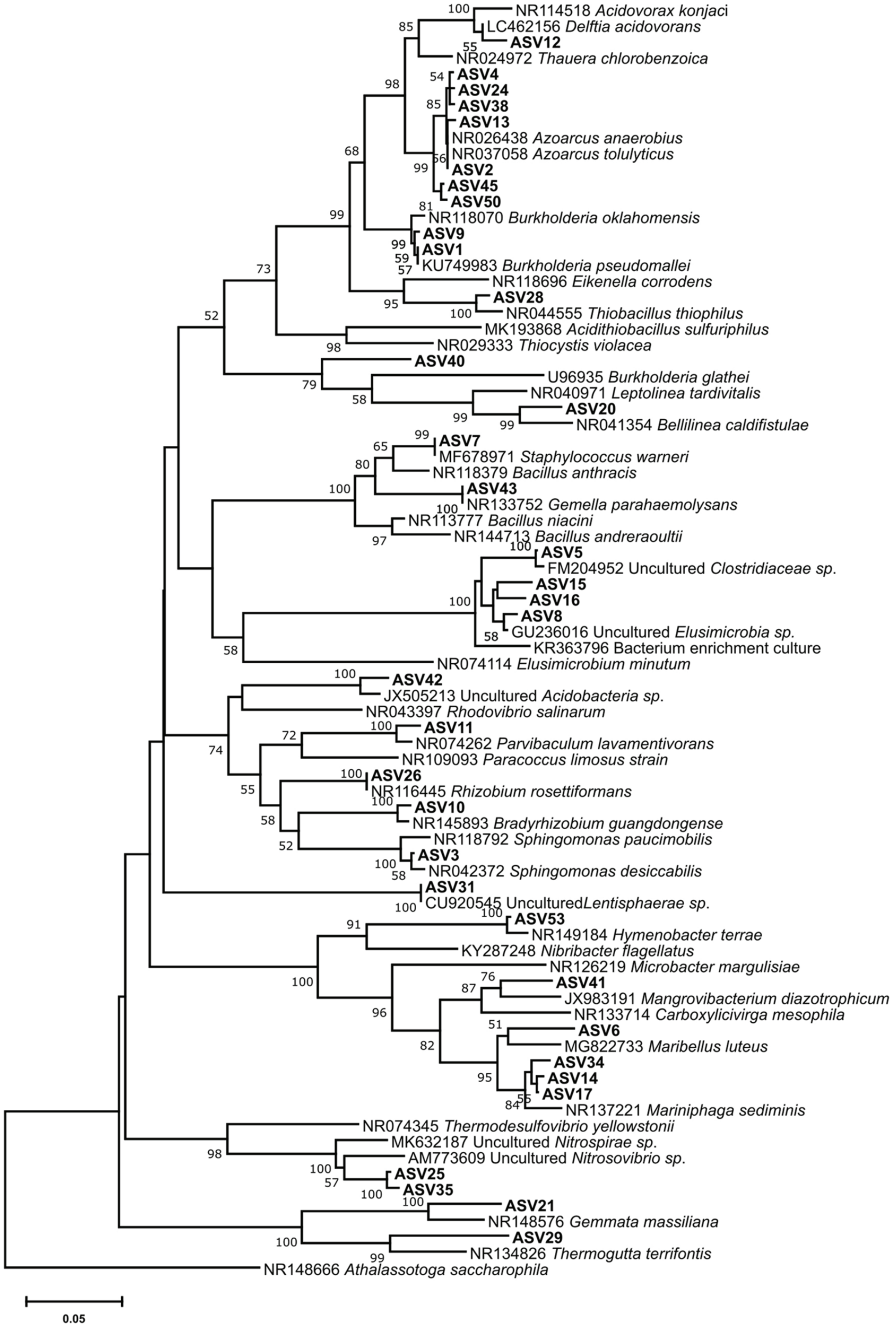
Bootstrapped neighbor-joining phylogeny of abundant (≥2%) ASVs and reference 16S rRNA sequences. Bootstrap support (1,000 replicates) >50% is indicated at the nodes. The scale bar represents 5% nucleotide sequence divergence. The phylogeny was calculated in MEGA-X based on the V4 region alignment using default parameters.

For each microcosm, more ASVs were detected in the light fractions than in the heavy ones. In the 8/0 and 5/7 microcosms, ASVs associated with members of the Zoogloeaceae were clearly enriched in the heavy DNA, accounting for up to 68 and 41% of the total community, respectively (Figure 3). Seven ASVs were found within the Zoogloeaceae, with five (ASV2, 4, 13, 24, and 38) representing *Azoarcus* spp., a well-known nitrate-reducing toluene degrader. The other two (ASV45 and 50) were from unclassified Zoogloeaceae. A phylogenetic tree was inferred for abundant ASVs, showing that the five *Azoarcus* ASVs were closely clustered, while ASV45 and 50 branched slightly more deeply (Figure 4). *Azoarcus* ASV4 was of very low abundance (0.1–0.4%) in all light fractions of ^13^C-toluene treatments. However, it accounted for about 25 and 12% in the heavy DNA of the 8/0 and 5/7 microcosms, respectively, but it was nearly absent (<0.1%) in the heavy DNA of the 0/18 microcosm with only nitrite as electron acceptor.

In the control and the 0/18 microcosms, ASV1 appeared most abundant in heavy DNA, accounting for up to *ca*. 76 and 52% of their total community, respectively (Figure 3). ASV1 and ASV9 both belonged to the Burkholderiaceae family and were closely related to *Burkholderia pseudomallei* and *Burkholderia oklahomensis* (Figure 4), both of which are not known for a capacity to degrade aromatic hydrocarbons (Limmathurotsakul et al., 2016). At the same time, ASV3, representing a species in the Sphingomonadales, was especially abundant in heavy fractions of the 5/7 and 0/18 ^13^C-microcosms, while was of very low abundance (<1%) in the corresponding light fractions. This lineage is known to host many toluene degrading bacteria, albeit mostly aerobic degraders (Kertesz et al., 2017).

## Discussion

Toluene can be oxidized to CO_2_ by many denitrifiers. Full oxidation of 1 mmol of toluene to CO_2_ yields 36 mmol of electrons, while the full reduction of 1 mmol of nitrate or nitrite to N_2_ requires 5 and 3 mmol electrons, respectively. Within the 1st week, about 0.5 mM toluene was oxidized in the 8/0 microcosm, while about 8 mM nitrate was reduced to nitrite, which was not further reduced during prolonged incubation (Figure 1). This can be explained by the fact that *Azoarcus* spp. were the dominant toluene-degraders in these microcosms (Figure 3), which have been reported to accumulate nitrite from nitrate under toluene limited conditions (Chee-Sanford et al., 1996). Nevertheless, based on the electron balance, toluene was fully oxidized to CO_2_ in these treatments.

In the 8/0 treatment, where no nitrite was provided, nitrate reduction did not proceed beyond nitrite. Intriguingly, when nitrate and nitrite were provided simultaneously (the 5/7 and 2/12 microcosms), nitrate appeared to be fully reduced within 6 days (Figure 1). In the 5/7 microcosm, nitrite but not nitrate appeared to be consumed initially, while nitrate reduction to nitrite started after the 3rd day, concurrent with a transiently increased nitrite concentration. However, in the end of incubation, nitrite concentration was similar to the initial value, indicating there was no net nitrite reduction. Possibly, this was observed since 5 mM of nitrate (by reduction to N_2_) is sufficient to fully oxidize 0.7 mM of toluene to CO_2_ (Figure 1). While in the 2/12 microcosm, nitrate alone was not enough to oxidize the toluene, so nitrite was also consumed along with nitrate depletion (Figure 1). Previous studies have indicated that nitrite vs. nitrate concentration and C/N ratio are important controls on nitrate/nitrite reduction pathways (Dong et al., 2009; Kraft et al., 2014). Although the end product of nitrate and nitrite reduction in these microcosms remained unclear, the progression of nitrate reduction was clearly influenced by the presence of nitrite. When nitrite was provided as the sole electron acceptor (the 0/18 microcosm), both nitrite reduction and toluene oxidation showed a long lag phase and the activity seemed to pick up only after day 6 (Figure 1). Presumably, the toxicity of nitrite in high concentrations could have caused this delay (Chayabutra and Ju, 2000; Ulrich and Edwards, 2003). Denitrifying hydrocarbon-degrading cultures have mostly been studied under nitrate reducing conditions to date (Widdel et al., 2010). The influence of nitrite availability, an obligate intermediate of nitrate reduction, on hydrocarbon degradation rates and degrader selection has hardly been investigated. Here, we demonstrate that degradation activities are clearly affected by nitrite. In further studies, more detailed and fine-tuned nitrite amendments could be tested to determine optimal combinations of nitrate and nitrite, which can improve total electron acceptor usage efficiency while avoiding nitrite toxicity.

Stable isotope probing is a powerful tool for dissecting active pollutant degraders in complex communities. Due to assimilation of heavier stable isotopes, genomes of the active degraders become enriched in heavy fractions of DNA gradients (Vogt et al., 2016). In our study, the community composition of ASVs in the heavy fractions of different treatments were clearly distinct (Figure 3), indicating that different degrader lineages were active in our treatments. In the 8/0 microcosm, *Azoarcus* spp. of the Zoogloeaceae within Betaproteobacteria were dominant and identified as the key toluene degraders (Figure 3). While in the 5/7 microcosm, with both nitrate and nitrite as electron acceptors, Zoogloeaceae as well as Sphingomonadales (ASV3) dominated the heavy fractions, both likely representing active key populations. Some members of the Sphingomonadales are known as aerobic, but not anaerobic aromatic hydrocarbon degraders, e.g., the recently described *Sphingobium aquiterrae* and *Sphingobium terrigena* (Revesz et al., 2018; Park et al., 2019). However, *Sphingomonas mucosissima*, which closely relate to ASV3 (Figure 4), has not been demonstrated for toluene degradation (Reddy and Garcia-Pichel, 2007). In our study, ASV3 microbes were among the most significantly labeled groups, suggesting an important role in denitrifying toluene oxidation. However, isolation and cultivation of an ASV3 bacterium will be needed to confirm its function in hydrocarbon degradation.

In both the 8/0 and the 5/7 microcosms, *Azoarcus* spp. were the dominant toluene degraders. However, the *Azoarcus*-related ASVs (ASV2, 4, 12, 24, and 38) were heterogeneously distributed. Relative abundance of *Azoarcus* ASV4, rather than ASV2, shifted strongly with changing electron acceptors. These ASVs are phylogenetically closely clustered together (Figure 4), and they would have been classified as one OTU when using the common 97% similarity cutoff. Thus, within-genus response to electron acceptor availability was only apparent *via* ASVs resolution. This further illustrates the advantage of using ASVs for environmental sequencing data analysis (Callahan et al., 2017).

Although ASV1, belonging to the *Burkholderiaceae*, was extremely abundant in the 0/18 microcosm heavy DNA, we do not assume it to be involved in toluene degradation. ASV1 also dominated heavy DNA of the control microcosm (Figure 3), where only ^12^C-toluene was provided. The genome of *B. pseudomallei*, closely related to ASV1 (Figure 4), is known to have a high G + C content of 68% (Holden et al., 2004). According to the linear relationship between DNA buoyant density (g ml^−1^) and G + C content (Buckley et al., 2007), ASV1 and *B. pseudomallei* DNA with a 68% G + C content, could have an intrinsic buoyant density of >1.72 g ml^−1^. This was similar to the density of heavy fractions, where ASV1 was found in the control microcosm. Thus likely, the appearance of ASV1 in heavy DNA was related to a G + C effect, rather than ^13^C-labeling. Very likely, ^13^C-labeling of active degraders would have been more clearly evident in SIP gradients loaded with 0/18 DNA extracted after 2 weeks of incubation. Unfortunately, this analysis was not conducted in the present work.

Distinct toluene-degrading microbial communities thus were labeled in microcosms with different electron acceptor amendments. The labeled groups were different to a previous DNA-SIP incubation probing toluene-degraders under hypoxic condition, albeit using sediments from the same site (Tancsics et al., 2018). *Quatrionicoccus* spp. were dominantly labeled under hypoxia, accounting for about 56% of all the bacterial community in the heavy fraction, followed by *Zoogloea* spp. (~14%) and *Rhodoferax* (~11%), while *Azoarcus* spp. were hardly labeled (Tancsics et al., 2018). This indicates that the Siklós aquifer harbors diverse respiratory groups of degraders, capable of functioning under various electron acceptor availability. Various microbes capable of denitrifying toluene degradation were isolated over the last decades, many of them affiliated to *Azoarcus* (Weelink et al., 2010). Recently, the genus *Azoarcus* has been reclassified from belonging to the previous Rhodocyclaceae to the new Zoogloeaceae family (Boden et al., 2017). The *Azoarcus* ASVs identified in our study closely relate to *Azoarcus anaerobius* and *Azoarcus tolulyticus* (Figure 4). However, based on the relatively short 16S rRNA reads from Illumina sequencing, a robust phylogenetic placement of these ASVs is not possible. Nevertheless, the differential pattern of nitrate and nitrite reduction observed in our microcosms could largely relate to different active degrader communities being stimulated and selected (Figure 3). The ratios of C/N and electron donors to acceptors could also have been important (Dong et al., 2009) and future studies should take this into account. Nonetheless, a small amendment of nitrite, in addition to nitrate, could activate indigenous microbes to more readily conduct full denitrification, resulting in a community capable of more efficiently using available electron acceptors. Therefore, providing both nitrate and nitrite to the contaminant degrading microbes could possibly increase bioremediation efficacy. Which ratios work the best, however, still requires further investigation.

Oxygenic denitrifiers are microbes proposed to form intracellular oxygen *via* nitric oxide dismutation. Previously, oxygenic denitrifiers have been suggested to be able to oxidize methane and long-chain alkanes, however, whether they can be involved in aromatic hydrocarbon degradation is still unknown (Zhu et al., 2017). It has been shown that oxygenic denitrifiers were present in the Siklós aquifer, accounting for a few percent of total bacterial gene counts in the sediment (Zhu et al., 2017, 2019). Transcripts of the nitric oxide dismutase (*nod*) gene, a functional marker for oxygenic denitrifiers were detected, albeit in extremely low numbers, in the labeled mRNA pool from a hypoxic microcosm, using Siklós aquifer sediment as inoculum and ^13^C_7_-toluene as substrate (Bradford et al., 2018). In the present work, the distribution of *nod* genes in SIP gradients indicated that oxygenic denitrifiers were also partially ^13^C-labeled. However, whether they directly degraded toluene or just assimilated metabolites from dominating degraders is not clear. Furthermore, *nod* abundance was very low, 5–6 orders of magnitude lower than that of 16S rRNA genes in respective DNA fractions. This indicates that oxygenic denitrifiers were not important in toluene degradation in these microcosms. Unlike the methane-driven oxygenic denitrifying NC10 bacteria, which have a strong preference for nitrite over nitrate (Ettwig et al., 2010), the abundance of labeled *nod* genes in our study did not show such a pattern. Further research for demonstrating the catabolic capacities of putative oxygenic denitrifiers is thus still needed.

## Conclusions

In this study, we demonstrated that nitrate and nitrite as electron acceptors influenced active toluene-degrading microbial populations in aquifer sediments. Members of the Zoogloeaceae were identified as key toluene degraders in microcosms with the presence of nitrate, while members of the Sphingomonadales were likely to be more important with increasing nitrite availability. Intra-genus variability of *Azoarcus* ASVs as a response to nitrate and nitrite was also demonstrated for the first time. The availability of nitrite also affected the outcomes of nitrate reduction. Our findings suggest a possibly overlooked beneficial role of nitrite in bioremediation. *Via* modulating active microbial communities, nitrite can enhance overall electron acceptor usage in sites, where nitrate is amended for pollutant degradation. This could offer new routes for optimizing future bioremediation strategies.

## Data Availability Statement

Sequencing data are available at NCBI with SRA accession: PRJNA631140.

## Author Contributions

BZ and TL designed the project. SF, BZ, and ZW performed the experiment. BZ analyzed the data with help from SF, ZW, AT, and TL. BZ wrote the manuscript with inputs from all authors. All authors contributed to the article and approved the submitted version.

### Conflict of Interest

The authors declare that the research was conducted in the absence of any commercial or financial relationships that could be construed as a potential conflict of interest.
